# Correction: Synthesis of 2-deoxy-2,2-difluoro-α-maltosyl fluoride and its X-ray structure in complex with *Streptomyces coelicolor* GlgEI-V279S

**DOI:** 10.1039/c5ob90111a

**Published:** 2015-07-02

**Authors:** Sandeep Thanna, Jared J. Lindenberger, Vishwanath V. Gaitonde, Donald R. Ronning, Steven J. Sucheck

**Affiliations:** Department of Chemistry and Biochemistry, The University of Toledo, 2801 W. Bancroft Street, MS602, Toledo, OH, USA 43606.

Correction for ‘Synthesis of 2-deoxy-2,2-difluoro-α-maltosyl fluoride and its X-ray structure in complex with *Streptomyces coelicolor* GlgEI-V279S’ by Sandeep Thanna *et al*., *Org. Biomol. Chem*., 2015, DOI: 10.1039/c5ob00867k.

The author regrets that some of the values given for the angles in the text in the two sentences beginning ‘The elongated bonding distances between α-MTF and Arg392 can be accounted for’ do not match with the values in Fig. 3.

The corrected sentences should read: The elongated bonding distances between α-MTF and Arg392 can be accounted for by a change in the *ϕ* torsion angle in the α-MTF (*ϕ* = 88.9° and *ψ* = 89.4°) relative to the MCP (*ϕ* = 88.1° and *ψ* = 74.2°) in the respective complexes. We suggest that this 15.2° change in *ψ* angle results from significant additional binding interactions afforded by the phosphonate moiety of MCP.

The Royal Society of Chemistry apologises for these errors and any consequent inconvenience to authors and readers.

